# Antioxidant Activity and Effectiveness of Fig Extract in Counteracting Carbon Tetrachloride-Induced Oxidative Damage in Rats

**DOI:** 10.3390/molecules29091997

**Published:** 2024-04-26

**Authors:** Leila Kebal, Noureddine Djebli, Katarzyna Pokajewicz, Nadjet Mostefa, Piotr P. Wieczorek

**Affiliations:** 1 Laboratory of Pharmacognosy and Api-Phytotherapy, Department of Biology, Faculty of Nature and Life Sciences, University of Mostaganem (UMAB), Mostaganem 2700, Algeria; 2 Department of Analytical Chemistry, Faculty of Chemistry and Pharmacy, University of Opole, Pl. Kopernika 11a, 45-040 Opole, Poland

**Keywords:** *Ficus carica* L., variety, antioxidant activity in vitro, in vivo, polyphenols, liver, free radical, HPLC-DAD

## Abstract

Figs are the edible fruits of the fig tree, *Ficus carica* L., that have been used for centuries for human consumption and in traditional medicine, to treat skin problems, inflammation, and gastrointestinal disorders. Our previous study investigated the presence of phenolic compounds in aqueous extracts of two Algerian popular fig varieties, *azendjar* (Az) and *taamriouth* (Ta), as well as their in vitro antioxidant activity. In this study, we assessed hydroethanolic extracts of these fig varieties. The total phenolic content was measured, along with the phenolic profile. Rutin was determined to be the dominant phenolic compound, followed by vanillic acid, 3,4-dihydroxybenzoic acid, quercetin, 4-hydroxybenzoic acid, rosmarinic acid (in Az only), and cinnamic acid. The antioxidant activity of the extracts was evaluated both in vitro (DPPH and FRAP assays) and in vivo, in rats intoxicated with carbon tetrachloride. In all assays, the fig extract—especially the dark-peeled fig variety *azendjar*—showed antioxidant potency. The administration of fig extract resulted in a reduction in liver damage, expressed by both different biochemical markers and histopathological study (less degraded liver architecture, reduced fibrosis, and only mild inflammation). A dose-dependent therapeutic effect was observed. The extract from the dark-peeled fig variety, Az, was characterized by a higher phenolic content and a stronger antioxidant activity than the extract from the light-peeled variety—Ta. Our study justifies the use of figs in traditional healing and shows the potential of using fig extracts in natural medicines and functional foods.

## 1. Introduction

Oxidation is fundamental to aerobic life, energizing living organisms for many biological processes [1]. The natural metabolism of oxygen releases reactive oxygen species (ROS) including non-radical species such as hydrogen peroxide (H_2_O_2_), hypochlorous acid (HOCl), singlet oxygen (^1^O_2_), and free radicals such as hydroxyl (OH^−^), peroxyl radicals (ROO*), and the superoxide anion (O_2_^−^) [2,3]. Free radicals can be generated by exogenous factors like air pollution, chemicals, ionic and non-ionic radiation, and toxic gases. At low concentrations, free radicals perform several beneficial biological functions such as helping phagocyte cells to destroy invading pathogens [1,3], whereas the presence of free radicals in high levels can generate oxidative stress, which leads to numerous dangerous disorders including tissue damage, hepatic and renal diseases, cardiovascular and degenerative problems, diabetes, and extensive lysis [4,5,6].

Several endogenic defense systems in a healthy living organism counteract oxidative stress and balance ROS and antioxidant systems. These antioxidant mechanisms can be enzymatic, including catalase, glutathione peroxidase, and superoxide dismutase enzymes. There are also non-enzymatic mechanisms like uric acid and glutathione [1,7]. When endogenous antioxidant defense mechanisms cannot completely prevent damage, the intake of exogenous antioxidants acting as free radical scavenging molecules is necessary. However, many synthetic antioxidant drugs have different side effects [1,5].

Dietary components are responsible for much of the human body’s antioxidant defense [8]. Nowadays, more emphasis has been placed on the intake of natural foods, which has prompted researchers to seek out bioactive substances with numerous benefits suitable for human consumption [9]. The consumption of fruits and vegetables can reduce the risk of many disorders caused by free radicals, such as cardiovascular diseases and cancer. In this case, bioactive compounds such as polyphenols, flavonoids, and vitamins were considered responsible for the antioxidant activity [4,10,11]. Polyphenols are the most important group of natural antioxidants [12]. Their antioxidant characteristics are caused by their redox properties, which allow them to operate as reducing agents and hydrogen donors [13].

*Ficus carica* L., commonly known as the fig tree and belonging to the Moraceae family, is one of the oldest cultivated trees in the world [14]. It is widely grown in the Mediterranean region, where its fruits are mainly consumed as food [15]. Besides their dietary usefulness, its fruits have long been used to treat various disorders, such as gastrointestinal, cardiovascular, respiratory, and inflammatory issues [16].

Fig fruits are a rich source of primary nutrients, including vitamins, carbohydrates, fatty acids, and minerals [17]. They were reported to contain the highest levels of antioxidant polyphenols among commonly available fruits in the diet [18]. These polyphenols are mainly located in the peel and are highly present in fruits with a dark peel color [15].

Algeria is a Mediterranean country and is known as one of the leading countries in the production of fig fruit for fresh and dried consumption, with more than 48 varieties of different peel colors. In a previous study, we examined the chemical composition of the aqueous extract of the two most famous varieties in the mountainous region of Algeria, *azendjar* and *taamriouth*, which have dark and light peel colors, respectively. The study confirmed that the Az variety, with a dark peel color, has a higher concentration of polyphenolics, compared to the Ta variety. The amount of rutin in the Az and Ta varieties of lyophilized fig extract was 84.16 ± 6.15 and 7.46 ± 0.76 µg/g, respectively, according to the HPLC-DAD analysis of the aqueous extract. The antioxidant potency of the aqueous extract of these two varieties was confirmed in vitro against DPPH and ABTS free radicals, as well as by using the FRAP technique [19].

The aim of this study is to analyze the chemical composition and antioxidant capacity of hydroethanolic extracts from fig fruits of the Az and Ta varieties, both in vitro and in vivo, against the oxidative damage caused by carbon tetrachloride in rats.

## 2. Results and Discussion

The present study is structured with dual objectives. The first aim was to investigate the phenolic compounds present in the extracts of both varieties of *F. carica* fruit for their antioxidant activity in vitro. The second aim was to validate the antioxidant capacity in vivo. The obtained results are presented below.

### 2.1. Phytochemical Identification of Plant Extract Using HPLC-DAD

**Figure 1:**
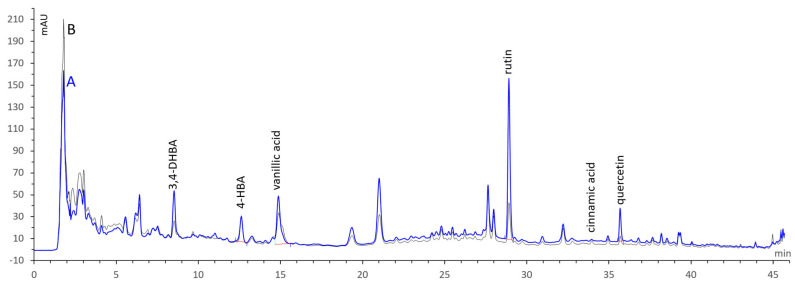
HPLC−DAD chromatograms (257 nm) of hydroethanolic extracts of *azendjar* (**A**, blue) and *taamriouth* (**B**, black) fig varieties.

Analyses were performed according to our previously published method [19]. Table 1 and Figure 1 presents the results obtained for the ethanolic extract of the studied fig fruits.

HPLC-DAD analysis revealed the presence of seven phenolic compounds. The phenolic acids detected in both extracts are 3,4-dihydroxybenzoic acid (protocatechuic acid), 4-hydroxybenzoic acid, and vanillic acid, with a dominant presence in the hydroethanolic extract of the Az variety, except for cinnamic acid, which was more abundant in the extract of the Ta variety, with 1.38 ± 0.13 µg/g of extract. Rosmarinic acid was found only in the extract of the Az variety, at a level of 0.87 ± 0.35 µg/g of extract. The flavonoids identified in the studied hydroethanolic extracts were rutin and quercetin.

The content of the phenolic compounds was higher in the extract of the Az variety with the dark peel color (Figure 1). In the limits of the standards, rutin was the major flavonoid presented in our *F. carica* fruit extracts, at levels of 140.64 ± 2.79 and 35.16 ± 0.27 µg/g of extract for the Az and Ta varieties, respectively, which is equivalent to 8.95 ± 0.17 mg/100 g DW and 2.54 ± 0.02 mg/100 g DW of fig fruit. Our previous study, investigating aqueous lyophilized extracts, confirmed the dominance of rutin in these two varieties, compared to the other phenolics. The hydroethanolic extract gave a higher amount than the aqueous ones, demonstrating amounts of 84.16 ± 6.15 and 7.46 ± 0.76 µg/g of aqueous extracts of the Az and Ta varieties, respectively [19]. These results are in line with those of Pereira et al. [20], who reported the amount of rutin as the major phenolic to range from 4.6 to 11.9 mg/100 g in the acetone extract of nine fig varieties, with the dark-colored varieties showing the highest levels.

Our results surpass those obtained by Faleh et al., [21] who reported values of rutin ranging from 2.363 ± 0.201 to 4.820 ± 0.32 mg/100 g DW in acidified aqueous extracts from 17 Tunisian fig cultivars. However, our values are lower than those found for the peel and pulp methanolic (80%) extract of figs, which registered as 18.2 and 9.5 mg/100 g FW, respectively [22]. The latter study also confirmed the dominance of rutin compared to other phenolic compounds. Studies by Soltana et al. [23], using LC-QTOF analysis of the methanolic extract of four Tunisian fig varieties, also demonstrated that rutin is the most dominant phenolic compound found in figs, with the highest amounts in the dark-peeled varieties (30.326 ± 2.178 mg/100 g), as opposed to the light-peeled ones (10.778 ± 0.39 mg/100 g). Several previous studies also reported that rutin is the major flavonoid found in figs [14,21,24]. Rutin has various pharmacological properties, including its vasoprotective, cytoprotective, cardioprotective, and neuroprotective potential. It is one of the most potent antioxidant agents used to treat and prevent cancer and neurodegenerative disorders [25,26,27].

Regarding quercetin, our results are higher than those of Faleh et al. [21], reporting amounts ranging from 0.057 ± 0.027 to 0.250 ± 0.036 mg/100 g DW. Quercetin is a flavonoid found in several plants and is known for its medicinal and pharmacological properties such as its anti-diabetic, anti-inflammatory, antioxidant, antimicrobial, anti-Alzheimer, anti-arthritic, cardiovascular, and wound-healing effects [28].

Concerning protocatechuic acid, our results are lower than those obtained by Viuda-Martos et al. [29], who found it at levels of 3.27 ± 0.03 and 6.22 ± 0.09 mg/100 g in the peel of fruits and 7.45 ± 0.04 and 8.11 ± 0.03 mg/100 g in the pulp of Spanish figs. Protocatechuic acid or 3,4-dihydroxy benzoic acid is known for its anti-inflammatory, anti-hyperglycemic, anti-ulcerous, antispasmodic, antioxidant, anti-cancer, anti-tumor, anti-aging, anti-atherogenic, anti-asthma, antibacterial, and neurological properties [30].

A study with HPLC analysis of three fig cultivars from Slovenia indicated several other phenolic compounds that were not detected in our extracts, such as gallic, syringic, and chlorogenic acid [31]. The difference in varieties can explain this, or, as reported in the literature, the amount of different bioactive chemicals in plants may be influenced by cultivation methods, geographical origin, and climatic and pedoclimatic conditions [32].

Many other analyses on the biochemical compounds of *F. carica* fruit extracts, performed using HPLC-DAD, have reported the presence of a large number of bioactive composites such as (-)-epicatechin, (+)-catechin [31], 5-O-caffeoylquinic acid, 3-O-caffeoylquinic acid [21], luteolin, apigenin, and catechin [29]. We intentionally avoided using the most efficient, but toxic, solvents to extract the most polyphenols from figs, in order to obtain safe extracts to test in vivo and, potentially, scale for the production of pharmaceutical raw materials.

In summary, our study confirms that *F. carica*. fruit extracts are a good source of bioactive compounds, which are responsible for the vast benefits of pharmacological activities.

### 2.2. Extraction Yields, Total Phenolic Content, and Antioxidant Activity In Vitro

The total phenolic content (TPC) was estimated using the Folin–Ciocalteu method, with gallic acid as the standard for the calibration curve. Th in vitro antioxidant activity was expressed as IC_50_ for the DPPH assay and as mmol Fe(II)/100 g of extract for the FRAP assay. All results are presented in Table 2.

#### 2.2.1. Extraction Yield

After the rotary evaporation, the extracts of the two varieties gave a pasty aspect, of dark brown and yellowish-brown color, with a yield of 10.15% and 9.95% for the Az and Ta varieties, respectively. Our results are in agreement with those of Soni et al. [33], who reported a yield of 11.47% for Indian fig extract, obtained with maceration in a mixture of four solvents (acetone, dichloromethane, ethyl acetate, and methanol). The extraction yield varies depending on the nature of the extract, the polarity of the extraction solvent, temperature, pH, and extraction time; it can even vary depending on the part of the plant subjected to extraction [34,35]. An aqueous mixture of acetone, ethanol, and methanol has been widely used to extract bioactive compounds from medicinal plants [36]. Bioactive phenolic compounds are ideally extracted using a mixture of water and ethanol for their extraction [37]. Mixtures of the appropriate organic solvent with water seem to be the best to obtain fractions rich in polyphenols [38]. This explains the use of the ethanol–water mixture in our hydroalcoholic extraction.

#### 2.2.2. Total Phenolic Content

The hydroethanolic extract of the Ta variety, with a light peel color, exhibited a lower TPC of 339.44 ± 10.33 GAE mg/100 g of extract, the equivalent of 245.57 ± 7.47 GAE mg/100 g DW of fig. This value was significantly different compared to the dark-peeled Az variety, which had a TPC of 403.66 ± 32.11 GAE mg/100 g of extract, equivalent to 257.06 ± 20.44 GAE mg/100 g DW of figs. The comparison of our results with the literature is hindered by differences in the solvent used, the unit of measurement, and the standard used for the calibration curve. It was reported that the mixture of water and alcohol can greatly affect the extractability of phenolic compounds, compared to monocomponent solvents [39]. Our findings are in agreement with those of Bachir bey and Louaileche [40], for the hydro-acetonic extract of nine varieties of fig fruits, including the Az and Ta varieties. The TPC ranged from 482.62 mg GAE/100 g to 644.11 mg GAE/100 g DW, with the highest value observed in dark fruits. Our TPC results are higher than those obtained for the methanolic (80%) extracts of five Albanian fig cultivars, where the total polyphenol content varied from 45.24 to 160.42 GAE mg/100 g DW [41]. The results of our present investigation are lower than those obtained for the ethanolic extract of South African fruits, reporting an amount of 104.67 ± 5.51 mg GAE/g of extract [42].

Although there are many differences in the amount of polyphenols present in figs, which are due to the type of solvent used for extraction [43] and the variety of fig [44], multiple studies have agreed that the dark-peeled fig varieties are richer in phenolic compounds than the light-peeled fig varieties [18,44].

#### 2.2.3. In Vitro Antioxidant Activity

- *DPPH assay*

The in vitro investigation of the antioxidant properties of plant extracts should include a DPPH assay for anti-free radical activity. The principle of the assay is the reduction of the radical 2,2-diphenyl-1-picrylhydrazyl (DPPH) to 2,2-diphenyl-1-hydrazine, in the presence of an antioxidant [45]. The results are expressed by calculating the inhibitory concentration (IC_50_) of the extracts compared to ascorbic acid, which was used as the standard, as mentioned in the Materials and Methods section. The lowest value of IC_50_ indicates the most potent ability of the sample to act as an antioxidant [44]. Both extracts exerted an antioxidant capacity against DPPH radicals, with the greatest effect exerted by the extract of the Az variety, with an IC_50_ of 0.417 ± 0.032 mg/mL, which was a highly significant difference from the Ta extract potency (0.582 ± 0.015 mg/mL). Our results are in line with those obtained by Palmeira et al. [46], who reported IC_50_ values of 0.46 ± 0.01 and 1.13 ± 0.05 mg/mL in 80% ethanolic extracts of the peel and pulp of a Portuguese variety of *F. carica* fruit. Our results are also supported by the results obtained for the ethanolic (70%) extracts of five cultivars of Croatian figs, reporting IC_50_ values ranging from 0.3947 to 0.7448 mg/mL [47]. The ethanolic extract of southern African figs showed a more potent antiradical activity against the DPPH radical, with an IC_50_ of 0.134 ± 0.018 mg/mL [42].

The literature supports the higher potency of the Az variety, with the dark peel, compared to the Ta variety, with the light peel. Aljane et al. [48] reported that the DPPH scavenging activity in purple–black ecotypes was more potent compared to that of the green ones, with average values of 50.25% and 26.95%, respectively. This was also confirmed by the study of the DPPH scavenging activity of Algerian fig varieties, which showed that the dark varieties have stronger antiradical activities against DPPH than the light ones, with mean values of 41.63 and 31.3%, respectively [40]. This was also supported by the results obtained from the methanolic extracts of five Albanian cultivars [41].

The antioxidant power of our extracts is expected to result from the presence of polyphenols, as proposed by Solomon et al. [49], who indicated that flavonoids and polyphenols are responsible for antioxidant activity. Phenolic matrices are a vibrant source of phytochemicals, which have many health benefits, including their ability to act as free radical scavengers [50].

- *FRAP assay*

Several factors make it difficult to compare our results with those reported in the literature, such as the different working protocols used, the different molecules used as standards, the different units of expression of the results, etc. However, this does not prevent us from highlighting the results of researchers who have studied the antioxidant activity of figs using the FRAP assay, which is the most striking and closest to our working conditions. The results obtained are expressed in mmol Fe_2_SO_4_/100 g per extract and per DW of figs. The hydroethanolic extract of the Az variety had a more potent FRAP activity, compared to the extract of the Ta variety, with a highly significant difference *p* ≤ 0.01 and values of 31.55 ± 1.43 mmol Fe (II)/100 g of extract and 26.08 ± 0.66 mmol Fe (II)/100 g of extract, respectively. The result obtained from the Az variety is in line with those obtained by the aqueous extract of the same variety in our previous study, which reported a value of 30.00 ± 3.25 mmol Fe (II)/100 g of the lyophilized extract, with *p* > 0.05. In comparison, the hydroethanolic extract of the Ta variety has shown a potent activity compared to the aqueous extract of the same variety, with a significant difference, which reported the value of 18.87 ± 2.62 mmol Fe (II)/100 g of the lyophilized extract [19]. Hssaini et al. [51] studied the FRAP assay of extracts from 11 fig varieties. Trolox was the standard used. The values of fig extracts ranged from 100 ± 4 to 1065 ± 18 mmol TE/100 g DW, confirming the potency of the dark peel color varieties compared to the light ones. The fig seeds extracted with 50% methanol had the highest antioxidant activities, when using the FRAP assay, with 8504 mg FeSO_4_/kg DW [43]. Pande and Akoh [52] reported FRAP test values ranging from 0.1 mM to 1.16 mM TE/100 g fresh fruit in brown figs, values which are inferior to our results.

Dark-peeled figs showed a valuable antioxidant potency, when using the FRAP assay, compared to light figs, which was supported in the literature [51].

### 2.3. In Vivo Antioxidant Activity

During or after the 14-day long administration of the hydroethanolic extracts studied, no behavioral abnormalities or deaths were observed in the test animals of the Az and Ta fig varieties at doses of 250, 350, 500, and at the highest dose of 2000 mg/kg bw.

#### 2.3.1. Variation of Body Weight

After 24 h of intraperitoneal injection (IP) of CCl_4_, a significant or even very highly significant weight loss, compared to the initial weight, was observed in all intoxicated groups, with growth rates ranging from −3.41% to −10.02%. This loss was followed by a weight recovery in all the batches treated with the extracts of the two varieties of figs and the group STD, treated with vitamin C, with growth rates ranging from −2.86 to 5.22% (Table 3). This weight recovery was impaired in group T+, which received no treatment, with a growth rate of −8.38% and ** *p* < 0.01, compared to the first day of the experiment. Our results are in agreement with those obtained by Truong et al. [53] and Dutta et al. [54], who highlighted the loss of body weight in CCl_4_-intoxicated mice. Decreased body weights from CCl_4_ intoxication are generally attributed to acute liver toxicity, evidenced by significant weight loss [55]. The oral administration of the hydroethanolic extracts of fig fruits and vitamin C restored weight gain. The *F. carica* extracts did not hinder the growth of the rats, which showed a continuous increase in body weight from the beginning of the treatment. The rats treated with the extracts demonstrated the ability to recover and regain weight, due to their efficiency in counteracting the induced hepatotoxicity, thus promoting the growth recovery of the rats. Such a correlation was also observed by Nemiche et al. [56] for nickel-intoxicated rats treated with the aqueous extract of the Ta variety of *F. carica* fruits.

#### 2.3.2. Determination of Biochemical Parameters

The determination of the hepatic biochemical parameters is presented in Table 4.

The results indicate that the intraperitoneal injection of carbon tetrachloride induced a highly significant (*p* ≤ 0.001) increase in serum transaminases (AST and ALT), bilirubin (BILT and BILD), and alkaline phosphatase compared with normal control rats, as well as treated rats. This is confirmed by several studies that have established the hepatotoxic effect of CCl_4_ on the serum liver parameters in rats [57,58]. These important metabolic enzymes are initially present in the cytoplasm of the liver cells and, in the event of liver disease, infiltrate into the bloodstream, in accordance with the extent of liver damage [59]. For this reason, increased blood levels of these enzymes serve as an indication of liver injury [60], confirming the hepatic damage caused by CCl_4_.

Treatment with fig hydroethanolic extracts reduces transaminases, bilirubin, and alkaline phosphatase in a dose-dependent manner. This indicates that these extracts protect hepatocytes against CCl_4_ and decrease intracellular enzyme leakage, thereby preserving the plasma membrane. Indeed, it has been reported that aqueous extracts, methanolic crude extracts, and *n*-hexane extracts of *F. carica* fruits decreased liver transaminases, ALP, and bilirubin in a dose-dependent manner in mice intoxicated with carbon tetrachloride [61], which is consistent with our results. Similarly, the aqueous extract of the Ta variety of figs induced a decrease in AST, ALT, and ALP in rats intoxicated by nickel [56]. In rats, serum AST and ALT levels decreased under pretreatment with *F. carica* fruit extract, in the gamma-induced hepatotoxicity model [62]. In addition, treatment with figs, as a diet, decreased serum transaminase levels (AST and ALT) against oxidative stress and ethanol-induced hepatotoxicity in rats [63].

The damage mechanism of CCl_4_ can be explained as oxidative damage caused by lipid peroxidation, which starts after the conversion of CCl_4_ to free radicals [64]. These reactive metabolites then damage the membranes of organelles and liver cells, leading to the release of cytosolic enzymes such as alanine aminotransferase and aspartate aminotransferase into the circulating blood [65]. The hepatoprotective effect of fig extracts seems to be due to the inhibitory nature of the phytochemicals they contain. According to Molehin et al. [66], phytochemicals are capable of inhibiting microsomal enzymes to restrict the generation of free radicals and to limit lipid peroxidation, through their antioxidant capacity. The effects exerted by the extracts of the fruits of *F. carica* are similar, or even better, to those exerted by ascorbic acid at a dose of 50 mg/kg bw, which has been used as a reference hepatoprotector. Ascorbic acid has been reported to normalize serum alanine and aspartate aminotransferase, alkaline phosphatase, and serum bilirubin levels in intoxicated animals. It is an important scavenger of free radicals in extracellular fluids and protects biomembranes from the damage caused by peroxides, by decreasing lipid peroxidation [67].

#### 2.3.3. Determination of Oxidative Stress Parameters

- Determination of tissue proteins

**Figure 2:**
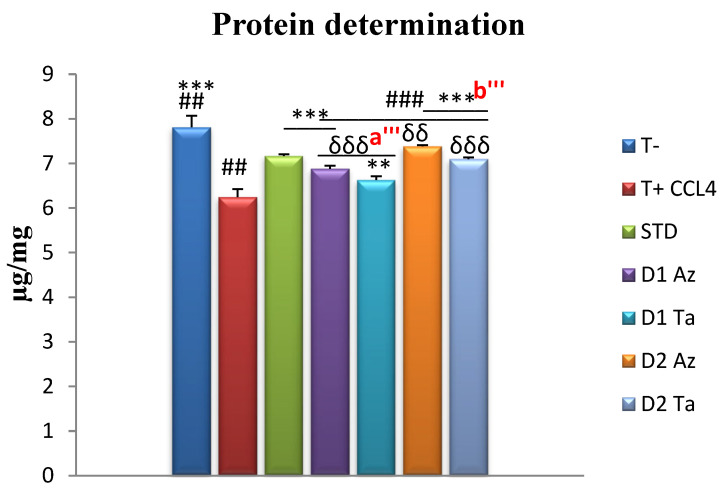
Hepatic protein assay, expressed in µg/mg tissue. Values are given as mean ± SEM (n = 5). Two symbols indicate *p* < 0.01; three symbols indicate *p* < 0.001 significantly different. Symbols: * compared to T+, # compared to STD, δ compared to T-, a’ is a comparison between groups treated with Dose 1 (300 mg/kg); b’ is a comparison between groups treated with Dose 2 (600 mg/kg).

The highest liver protein content was marked in the T- group (7.81 ± 0.25 µg/mg of tissue), with a significant difference from the other groups (Figure 2). In contrast, the T+ group had the lowest protein content (6.24 ± 0.17 µg/mg of tissue), with *** *p* ≤ 0.001, compared to all other groups. All groups treated with the first dose of both extract varieties marked values lower than the standard (7.16 ± 0.03), while the groups treated with the second dose had higher values, with *** *p* ≤ 0.001. It is noted that the treatment with the Az extracts marked the levels of liver tissue protein closest to the negative control. The Dose 2 group, treated with the 600 mg/kg bw dosage, had higher values than the Dose 1 (300 mg/kg bw) group.

Our study showed a highly significant decrease in liver tissue protein levels following CCl_4_ intoxication. At the same time, there was a remarkable dose-dependent increase when treated with the plant extracts and the vitamin C used as the standard. Our results agree with several recent studies investigating plant extracts’ effect on tissue protein levels, following CCl_4_-induced intoxication in the liver [68,69,70].

Carbon tetrachloride can induce cellular hypo-methylation, inhibiting protein synthesis (possibly through the hypo-methylation of ribosomal RNA) and abnormalities in lipid and protein metabolism [71]. CCl_4_ is transformed into free radicals. The production of ROS can damage the cell by altering the fluidity of membranes and damaging lipids, proteins, and DNA; free radicals such as hydroxyl radicals can damage proteins and, consequently, can lead to various results in various pathophysiological disorders [68].

- Determination of malondialdehyde (MDA)

MDA is one of the end products formed during the decomposition of polyunsaturated fatty acids by free radicals [72]. It is the most widely used marker to study lipo-peroxidation.

Hepatic MDA levels in the negative control rats not poisoned with CCl_4_ were 43.66 ± 10.59 nmol/g tissue, a highly significant difference compared to other groups. Treatment with *F. carica* extracts at 600 mg/kg bw exerted a significant MDA-lowering effect, compared with the standard group (85.45 ± 1.16 nmol/g tissue). Extracts exerted a dose-dependent effect, with the Az variety extracts being more potent than the Ta variety extracts. The intraperitoneal injection of CCl_4_ induces a highly significant increase in hepatic MDA levels, increasing lipid peroxidation. Our results were in agreement with several studies that have addressed lipoperoxidation in rats following CCl_4_ intoxication [73,74]. The hepatotoxic effects of carbon tetrachloride metabolism are due to its metabolite, the trichloromethyl radical, which binds covalently to macromolecules and induces the peroxidative degradation of endoplasmic reticulum membrane lipids rich in polyunsaturated fatty acids. This leads to the formation of lipid peroxides, which, in turn, produce a product such as MDA. The process of the peroxidation of biomolecules is the leading cause of the hepatotoxicity of CCl_4_. The increase in hepatic MDA levels in CCl_4_-treated rats suggests that the natural antioxidant defense mechanism for scavenging excessive free radicals has been compromised [75].

**Figure 3:**
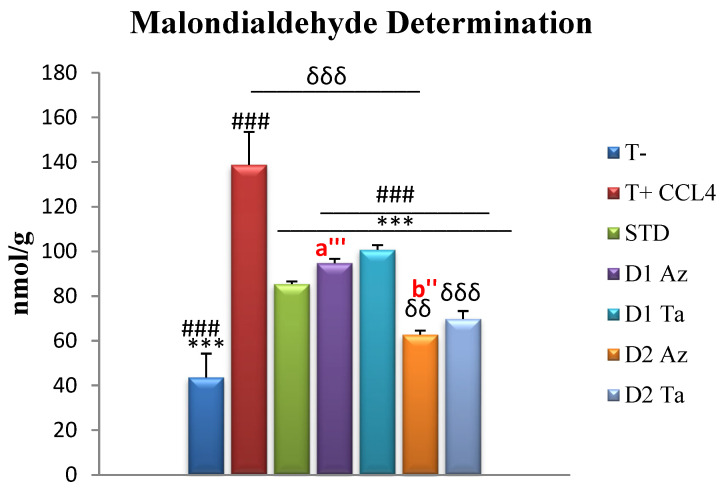
Hepatic MDA assay expressed in nmol/g tissue. Values are given as mean ± SEM (n = 5). Two symbols indicate *p* < 0.01; three symbols indicate *p* < 0.001 significantly different. Symbols: * compared to T+, # compared to STD, δ compared to T-, a’ is a comparison between groups treated with Dose 1 (300 mg/kg); b’ is a comparison between groups treated with Dose 2 (600 mg/kg).

As shown in Figure 3, both extracts of the fruits significantly reduced MDA levels in liver homogenates, in a dose-dependent manner. This effect of decreasing MDA levels has been supported in several studies investigating the efficacy of extracts from *F. carica* fruits in mitigating the elevation of MDA levels induced by various toxic agents [56,63].

Our in vitro study showed that the phenolic compounds in *F. carica* fruits are important antioxidants. Furthermore, these results supported the beneficial effect of *F. carica* in maintaining the integrity and function of hepatocytes. These effects may be due, at least in part, to its antioxidant activity.

Almost all phenols can act as lipid peroxidation antioxidants because they scavenge the chain-carrying lipid peroxyl radicals [76,77]. Rudrapal et al. [78] mentioned some phenolic compounds that act against the accumulation of lipid peroxidation products including quercetin, which we found in our extracts. Rutin, the major flavonoid in our extracts, showed a promising effect in the significant inhibition of MDA levels in the liver tissue of CCl_4_-intoxicated rats [74].

- Determination of catalase (CAT)

**Figure 4:**
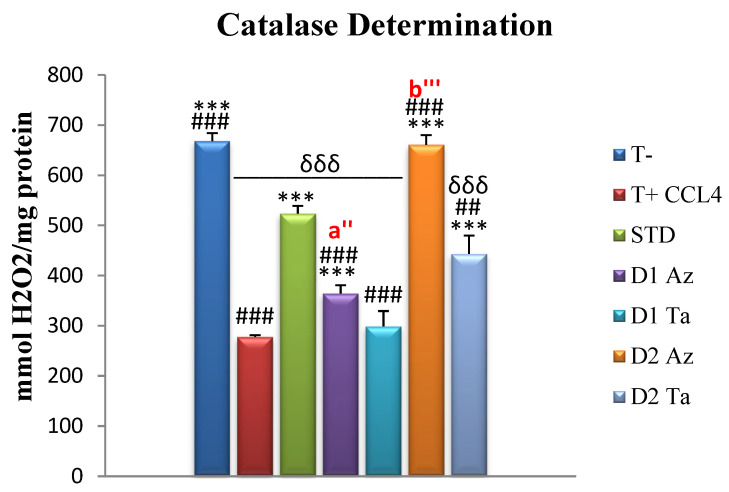
Hepatic catalase levels assay expressed in mmol H_2_O_2_/mg protein. Values are given as mean ± SEM (n = 5). Two symbols indicate *p* < 0.01; three symbols indicate *p* < 0.001 significantly different. Symbols: * compared to T+, # compared to STD, δ compared to T-, a’ is a comparison between groups treated with Dose 1 (300 mg/kg); b’ is a comparison between groups treated with Dose 2 (600 mg/kg).

Hepatic tissue catalase levels are expressed in mmol H_2_O_2_/mg protein and are presented in Figure 4.

The lowest catalase levels were recorded in positive control rats, T+, (277.72 ± 3.49), with *** *p* ≤ 0.001, compared to other groups. The control group, T-, that was not intoxicated scored an average of 668.25 ± 15.76, with a significant difference from the other groups, except the Az group treated with Dose 2 (*p* > 0.05). Compared to the standard group, STD, treated with vitamin C, treatment with extracts at 600 mg/kg bw showed a better effect, while treatment with 300 mg/kg bw gave a lower impact than the standard (with *** *p* ≤ 0.001). CAT activity is a commonly used test that can provide information about the cell’s antioxidant defense system. Catalase, an enzymatic antioxidant, circulates widely in all animal tissues, with the highest activity found in red blood cells and the liver [79]. Catalases are enzymes that help the cell defend against oxidative stress by neutralizing hydrogen peroxide [80]. The reduced activity of these enzymes, therefore, leads to a number of adverse effects, due to the accumulation of hydrogen peroxide and superoxide radicals [79].

In this study, the CCl_4_ intoxication of rats resulted in reduced CAT activity in the liver. This is in agreement with several previous studies that have addressed the determination of catalase in tissues following intoxication [68,69,74]. The administration of *F. carica* extracts enhanced CAT activity in rats with CCl_4_-induced liver injury to prevent the accumulation of unwanted free radicals and to protect the liver from the effects of intoxication. Our results are supported by those of Hira et al. [61], who reported the dose-dependent CAT-enhancing activity of *F. carica* extracts.

The power of *F. carica* extracts to improve CAT levels is due to their richness in antioxidant phenolic compounds. Several phytochemicals, such as rutin, salidroside, and naringenin, have been reported to have catalase-stimulating activity in rats, following CCl_4_ intoxication [64]. Some other studies showed that the administration of polyphenol-rich extracts significantly increased the activity of antioxidant enzymes, notably catalase [54,81,82].

#### 2.3.4. Histological Study

**Figure 5:**
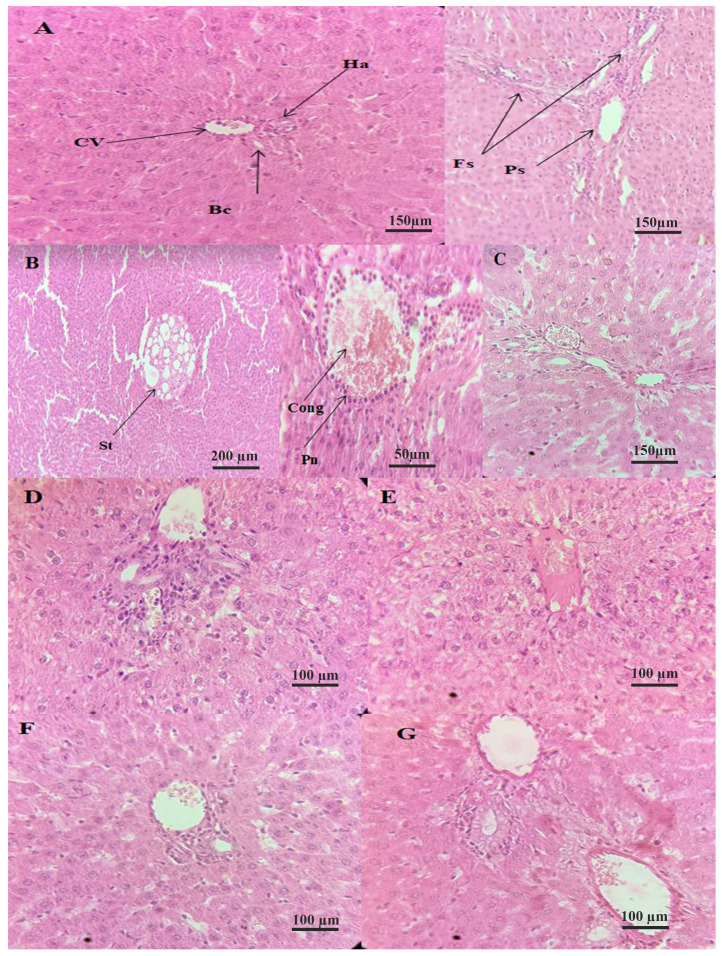
Histology sections of hepatic tissues (HE) × 40 (**A**): negative control group, (**B**): positive control group, (**C**): standard group (Vit C), (**D**): treated group with the Az *F. carica* extract at 300 mg/kg, (**E**): treated group with the Ta *F. carica* extract at 300 mg/kg, (**F**): treated group with the Az *F. carica* extract at 600 mg/kg, and (**G**): treated group with the Ta *F. carica* extract at 600 mg/kg. cv: centrilobular vein; Ha: hepatic arteriole; Bc: bile duct; Fs: fibrous septa; Ps: portal space; St: steatosis; Cong: congestion; Pn: polynuclear.

The histological study of the liver highlighted several aspects, as shown in Figure 5.

The microscopic appearance of liver tissue from healthy rats (T-) presents a normal physiological appearance with well-structured and delineated centrilobular veins, biliary ducts, and portal spaces. Several studies that focused on the histological study of rat liver tissue presented the same aspect [83,84,85].

Microscopic examination of the liver tissue from CCl_4_-intoxicated rats in the positive control group (T+) reveals the destruction of the tissue architecture (A in Figure 5), expressed by the presence of polymorphs in the centrilobular vein, inflammatory infiltrates, congestion in the centrilobular vein, and macrovascular hepatic tissue steatosis. The exact appearance of liver tissue in rats intoxicated with CCl_4_ at the same dose and protocol was also observed by Ojeaburu and Oriakhi [86], where the observation of large intra-cytoplasmic fat vacuoles (macrovesicular steatosis), vascular congestion, and extensive periportal inflammatory infiltrates were reported.

The liver tissue from rats in the standard group (STD) showed moderate congestion of the centrolobular vein, general preservation of the liver tissue, and a mild inflammatory infiltrate, compared with T+. The liver tissue from CCl_4_-intoxicated rats treated with vitamin C at 300 mg/kg bw showed reduced vacuolization compared to T+, small vacuoles, and fewer mitotic hepatocytes than T+ [87].

The tissues from rats treated with 300 mg/kg of the Az variety extract exhibited a less intense moderate inflammatory infiltrate compared to the positive control, with dilatation of the sinusoids and portitis lesions. In contrast, tissues from rats treated with Dose 1 of the Ta variety extract showed dilatation of the centrolobular vein and moderate disruption of tissue architecture. Within the limits of the fragments studied, the treatment of rats with the 300 mg/kg dose showed a marked hepatoprotective effect compared to T+ and was similar to that of STD, which was more pronounced in rats treated with extracts of the Az variety.

The liver tissue from rats treated with the Az variety extract at a higher dose (Dose 2)—600 mg/kg bw—showed general tissue preservation, a slight inflammatory infiltrate organized in clusters, and slight congestion of the centrolobular vein. A similar observation was carried out for rats treated with the Ta variety (Dose 2), with a slight dilation of veins and inflammation of the portal spaces. The preservation was dose-dependent. The histological study of the liver revealed that rats treated with a higher dose of 600 mg/kg bw had less damaged liver than those treated with 300 mg/kg. This dose-dependent hepatoprotective effect of fig extracts was also observed by Hira et al. [61]. They reported that the administration of methanolic, *n*-hexane, and aqueous extracts of *F. carica* fruits reversed the gross damages observed in the liver cell architecture, in a dose-dependent manner. Even in the case of hepatotoxicities induced by other hepatotoxic agents, fig fruits have shown their hepatoprotective action on the histological scale. Nemiche et al. [56] demonstrated nickel-induced oxidative damage on the histological level of the liver. At the same time, the administration of fig extract improved the restoration of liver alterations in rats. Furthermore, it was reported that consuming figs in a diet had a hepatoprotective effect against ethanol-induced hepatotoxicity, where histological sections showed an absence of hydropic degeneration, necrosis, coagulation in hepatocytes, and fibrosis [63].

The structure of the livers of rats treated with *F. carica* extracts became more similar to the liver structure of the healthy subjects, as the dosage increased. This demonstrates that treatment with these extracts could accelerate the ability of liver cells to regenerate after CCl_4_-induced oxidative stress. This finding is consistent with what Turan and Celik [63], as well as Nemiche et al. [56], have reported in their studies. 

In light of all the above, the present study justifies the use of figs in traditional medicine and their current use as nutraceuticals, by demonstrating its richness in various antioxidants, which, when consumed, contribute to reducing the risk of several disorders related to oxidative stress. The main future purpose of our study is to develop new treatments based on natural medicinal plant materials with fewer side effects than artificial ones. Figs are a promising fruit in the future of natural medicine and functional foods.

## 3. Materials and Methods

### 3.1. Reagents

The phenolic compounds were sourced from Sigma-Aldrich (Merck, Darmstadt, Germany). The standards set included gallic acid monohydrate (5995-86-8), 3,4-hydroxybenzoic acid (99-50-3), 4-hydroxybenzoic acid (99-96-7), chlorogenic acid (327-97-9), vanillic acid (121-34-6), caffeic acid (331-39-5), 3-hydroxybenzoic acid (99-06-9), syringic acid (530-57-4), trans-ferulic acid (537-98-4), rutin hydrate (207671-50-9), ellagic acid (476-66-4), rosmarinic acid (20283-92-5), cinnamic acid (140-10-3), quercetin dihydrate (6151-25-3), and chrysin (480-40-0). Methanol (67-56-1) of HPLC grade (Riedel de Haen) and formic acid (64-18-6) of LC-MS grade (Fluka) were obtained from Honeywell (Seelze, Germany). Water (7732-18-5) of HPLC grade was generated in-house, using a Hydrolab SPRING water treatment station (Hydrolab, Straszyn, Poland). Carbon tetrachloride (65-23-5), used for the in vivo study, was from Merck. For the histological study, the formaldehyde solution (50-00-0) was from Biochem Chemopharma (Cosne-Cours-Sur-Loire, France). Acetone (67-64-1) and toluene (108-88-3) were from Sigma-Aldrich. Hematoxylin (517-28-2) and eosin (548-24-3) were from Prochima-sigma (Tlemcen, Algeria). For the total phenolic compounds determination, Folin–Ciocalteu reagent (FCR) and gallic acid monohydrate (5995-86-8) were from Sigma-Aldrich. Sodium carbonate (497-19-8) was from Fluka. The in vitro antioxidant assay reagents such as 2,2-diphenyl-1-picrylhydrazyl (1898-66-4), 2,4,6-tris(2-pyridyl)-s-triazine (3682-35-7), iron(II) sulfate heptahydrate (7782-63-0), and sodium acetate (127-09-3) were from Sigma-Aldrich. Ferric chloride (7705-08-0) was from VWR chemicals (Avantor, Radnor, PA, USA). Ethanol (64-17-5), methanol (67-56-1), and hydrochloric acid (7647-01-0) were from Riedel-de Haën (Honeywell, Charlotte, NC, USA). Sodium chloride (7647-14-5) 0.9% was from Biolyse laboratory (Rouiba, Algiers, Algeria). Ascorbic acid (50-81-7) was from Sigma-Aldrich. Kits used for the determination of the various biochemical parameters were from EliTechGroup Sées (Sées, France). For the study of the oxidative stress enzymatic parameters, potassium chloride (7447-40-7), bovine serum albumin (9048-46-8), trichloroacetic acid (76-03-9), 2-thiobarbituric acid (504-17-6), 1,1,3,3-tetramethoxypropane (102-52-3), hydrogen peroxide solution (7722-84-1), Coomassie Brilliant blue G 250 (6104-58-1), and *n*-butanol (71-36-3) were from Sigma-Aldrich.

### 3.2. Plant Samples

Fruit specimen varieties were first identified by experts of ITAFV (Technical Institute for Fruit Trees in Algiers, Algeria). The fruits were harvested from Aghbal (36°30′10″ N 1°50′45″ E, Tipaza, north of Algeria). The specimens are known as the *azendjar* (Az) and *taamriouth* (Ta) varieties and have dark and light peel colors, respectively. Voucher specimens were placed at the herbarium of the Department of Biological Sciences of Mostaganem University (Mostaganem, Algeria) (UM/09/2020).

### 3.3. Drying Procedure

The fruits were arranged in a layer and exposed to sunlight for ten days while being protected from humidity [88]. The figs were cut into small pieces, lyophilized under vacuum for 48 h, and then crushed into a fine powder. The powder was stored in shaded, sealed glass containers at −20 °C until further use.

### 3.4. Crude Extract Preparation

A hydro-ethanolic extract was prepared, following the method reported by Gilani et al. [89]. The fine powder was macerated in a mixture of 80% ethanol in distilled water (8:2/V/V), in the proportion of 25 g of powder per 100 mL of solvent, with magnetic stirring (300 rpm) at 24 ± 4 °C for 72 h. The resulting homogenate was double-filtered through a muslin cloth (0.25 mm mesh size) and Whatman filter paper N°1. The extraction was repeated and the resulting filtrates were combined. The solvent of the filtrate was evaporated under a vacuum, with a rotary evaporator, at 45 °C. The residue was a thick paste of dark brown and yellow-brown color for the Az and Ta varieties, respectively. The extracts were kept in the fridge at −20 °C until further use. The percentage yields of the extracts were calculated as follows [90].

$$\mathrm{Extract}\;\mathrm{yield}\;\mathrm{percentage}=\frac{\mathrm{weight}\;\mathrm{of}\;\mathrm{extract}}{\mathrm{weight}\;\mathrm{of}\;\mathrm{plant}\;\mathrm{powder}}\times 100$$

### 3.5. HPLC-DAD of Fruit Extract

The extracts were analyzed in triplicate using an in-house method developed by the authors, with a Dionex Ultimate 3000 system (Thermo Fischer, Carlsbad, CA, USA) and a diode array detector (DAD). The column used was C18 and the eluent was a mixture of methanol and 0.1% formic acid in water. The exact analysis conditions and analytical parameters of the developed method are presented in our previous article [19].

For this study, 14 phenolic compounds were used as standards, as follows: gallic acid, 3,4-hydroxybenzoic acid (3,4-HBA; protocatechuic acid—PCA), 4-hydroxybenzoic acid (4-HBA; *p*-hydroxybenzoic acid—PHBA), chlorogenic acid, vanillic acid, caffeic acid, 3-hydroxybenzoic acid (3-HBA, *m*-hydroxybenzoic acid), syringic acid, ferulic acid, rutin, rosmarinic acid, cinnamic acid, quercetin, and chrysin. The identification of the compounds was based on retention time, UV absorption spectrum, and the addition of standards to the analyzed extract samples. Results are expressed as µg/g of extract and in mg per 100 g of fig DW (dry weight).

### 3.6. Total Phenolic Content (TPC)

Total phenolic content was estimated according to the method described by Singleton and Rossi [91]. Briefly, 200 µL of the extract was added to an 800 µL Folin–Ciocalteu reagent (FCR) (diluted in distilled water at a ratio of 1/10), before 1 mL of a mixture of 7.5% (*w*/*v*) sodium carbonate was added. The absorbance of the mixture was measured at 765 nm, after incubation for one hour at room temperature, against a reagent blank. Gallic acid was used as a standard for establishing the calibration curve (R^2^ = 0.999).

### 3.7. In Vitro Antioxidant Activity

The antioxidant capacity of the fig varieties was evaluated in vitro using the DPPH radical scavenging method and their ability to reduce compounds by donating electrons (FRAP method).

#### 3.7.1. DPPH Assay

The DPPH (2,2-diphenyl-1-picrylhydrazyl) scavenging activity was determined as follows: A mixture of 2 mL, containing 50 µL of the extract and 1950 µL of DPPH solution (60 µM) prepared in methanol, was prepared and kept at 23 ± 2 °C for 30 min. Absorbance was measured at 515 nm against a control containing the extraction solvent and DPPH reagent only. A calibration curve was constructed with ascorbic acid and was used as a positive control for quantifying the antioxidant activity.

The percentage of inhibition of DPPH radical or radical scavenging activity (RSA) was calculated as follows:

$$RSA\left(\%\right)=\frac{\left(\mathrm{Abs}\;\mathrm{control}-\mathrm{Abs}\;\mathrm{sample}\right)}{\mathrm{Abs}\;\mathrm{control}}\times 100$$

RSA—otherwise known as IC_50_ or 50% inhibitory concentration—is the concentration responsible for scavenging 50% of DPPH radical (sample concentration range from 0 to 1 mg/mL) [92].

#### 3.7.2. Ferric Ion Reducing Antioxidant Power (FRAP)

The assay was performed as follows [93,94]: FRAP reagent was freshly prepared by mixing three stock solutions of sodium acetate buffer (0.3 M, pH = 3.6), 20 mM of FeCl_3_ (in demineralized water), and 10 mM of 2,4,6-tris(2-pyridyl)-s-triazine (TPTZ) in a 40 mM HCl solution, in a ratio of 10:1:1. The mixture was warmed in a water bath for 6 min. In total, 50 µL of each extract or standard solution was added to 1500 µL of FRAP reagent. The mixture was incubated for 5 min at 23 ± 2 °C. Absorbance was measured at 594 nm against the control. Iron (II) sulfate heptahydrate was used as the standard for the calibration curve and the results were expressed as micrograms of ferrous sulfate equivalents per gram of DW.

### 3.8. Determination of the Antioxidant Activity In Vivo

A total of 75 Wistar female rats (*Rattus norvegicus domestica*), weighing approximately 180 ± 15 g, were purchased from the Pasteur Institute Kouba, Algiers, Algeria; housed in polypropylene cages (21.5 × 15.5 × 8 feeder); and transported to the animal research facility of the Mostaganem University, Algeria. The rats were kept in a specific-pathogen-free environment and under controlled conditions, as follows: temperature (22 ± 2 °C) and light cycle system (12 h darkness, 12 h brightness). The acclimatization period was 14 days, with ad libitum access to water and food, which were dry pellets obtained from the National Livestock Feed Office (N.L.F.O) in Bouzaréat, Algeria. The in vivo studies were conducted according to the European Communities Council Directive (2010/63/EU) for animal experiments and the protocol employed was approved by the Ethical Committee (University of Mostaganem) under the inscription number 01/LPAP-SNV/23.

#### 3.8.1. Acute Toxicity Test (ATT)

The test was performed according to guideline 425 of the Organization for Economic Cooperation and Development (OECD) [95]. Forty rats were divided into eight groups (A to H) of five rats each, with similar body weight and received, via intragastric gavage (IGG), the doses of Az and Ta hydroethanolic fig extract, respectively, at 250 mg/kg (body weight) (for A and B groups); 350 mg/kg body weight (bw) (for C and D groups); 500 mg/kg bw (for E and F groups); and 2000 mg/kg bw (for G and H groups). The rats were observed for any signs of toxicity, such as convulsions, diarrhea, and increased locomotion, after the first 6 h of extract administration and then daily for 14 days [96].

#### 3.8.2. In Vivo Antioxidant Activity

Thirty-five rats were divided into seven groups of similar body weight (n = 5), with the first group serving as a negative control that received no intoxication or treatment; groups 2 to 7 were intoxicated with a single dose of CCl_4_ (1.25 mL/kg in olive oil (1:1); IP) to induce hepatic damage [86]. After the CCl_4_ injection, rats were subjected to daily intragastric gavage (IGG) for seven days with distilled water for groups 1 and 2 as negative and positive control groups (healthy and intoxicated with no treatment, respectively); ascorbic acid (vitamin C) at 50 mg/kg bw dissolved in 0.9% NaCl as a standard group 3; extracts of Az and Ta varieties at a dose of 300 mg/kg bw, respectively, for groups 4 and 5; and extracts of Az and Ta varieties at a dose of 600 mg/kg bw, respectively, for groups 6 and 7.

#### 3.8.3. Monitoring of Rat Body Weight

The body weight of the rats was monitored on a regular and daily basis. It was measured in grams (g), with a Scaltec SBA 52 balance, and the growth rate of the rats from day one was expressed as a percentage and calculated according to the following formula [97]:

$$\mathrm{Growth}\;\mathrm{rate}\left(\%\right)=\frac{\left(Wd-Wd\;0\right)}{Wd\;0}\times 100$$

where Wd is the weight on the day of weighing and Wd 0 is the weight on the first day of the study.

#### 3.8.4. Biochemical Analysis

On day 8, a blood sample was collected from the rats, who were slightly anesthetized, via a puncture in the retro-orbital sinus at the level of the eye, using capillary tubes with microhematocrit. The blood was collected in tubes containing an anticoagulant (0.1% heparin) and was centrifuged at 2000 rpm for 10 min using a HETTICH model Rotofix 32 centrifuge. The serum obtained was used to determine the hepatic enzymes such as alanine aminotransferase (ALT), aspartate aminotransferase (AST), and alkaline phosphatase (ALP), as well as total bilirubin (BILT) and direct bilirubin (BILD) [98,99,100,101,102]. The rats were euthanized and a ventromedial dissection was performed; the liver was removed and rinsed with ice-cold physiological water and divided into two parts; one part was used for histological study and the other part was stored at −86 °C for the study of oxidative stress enzymatic parameters.

### 3.9. Tissue Assay of Oxidative Stress Parameters

#### 3.9.1. Preparation of the Cytosolic Fraction of Tissues

A total of 1 g of liver was added to 9 mL of potassium chloride solution (0.15 M) at 4 °C. The mixture was homogenized with a Dounce homogenizer and then centrifuged at 3000 rpm for 20 min at 4 °C [103].

#### 3.9.2. Determination of Tissue Proteins

Proteins were determined according to the Bradford technique [104]. Briefly, 50 µL of the cytosolic fraction was added to 2 mL of Coomassie Blue Reagent. The mixture was vortexed and incubated for 5 min at 23 ± 2 °C. Absorbance was measured at 595 nm against a blank. Bovine serum albumin (BSA) was used as a standard, under the same conditions, for a calibration curve, with concentrations ranging from 0 to 1 mg/mL.

#### 3.9.3. Malondialdehyde (MDA) Determination

In total, 0.5 mL of the tissue homogenate, 0.5 mL of trichloroacetic acid (TCA 20%), and 1 mL of thiobarbituric acid (TBA) 0.67% were mixed. The mixture was vortexed and incubated in a water bath at 100 °C for 15 min. The mixture was then cooled and 4 mL of *n*-butanol was added. After centrifugation for 15 min at 3000 rpm, the supernatant was collected and the optical density was measured at 532 nm, against a blank. The calibration curve was prepared under the same conditions with 1,1,3,3-tetramethoxypropane concentrations ranging from 0 to 10 nmol. Results are expressed as nmol/g tissue [105].

#### 3.9.4. Cytosolic Catalase (CAT) Determination

A 1 mL reaction mixture contained 780 µL phosphate buffer (pH = 7.4, 0.1 M), 200 µL hydrogen peroxide (0.5 M), and 20 µL sample homogenate. Absorbance was measured at 240 nm. Results are expressed as mmol H_2_O_2_/mg protein, using the following equation: [80,106].

$$CAT[mmol\;H_{2}O_{2}/min/mg\;Pro)=\frac{\left(\Delta Do\right)}{\varepsilon \times W\times Y\;mg\;protein}$$

where

ΔDo: Delta of absorbance measurements

ε: Molar linear extinction coefficient = 0.0436 mM

W: Width of the measuring vessel in cm = 1 cm

Y: Protein content in mg/mL

### 3.10. Histological Analysis

The histological study was effectuated according to the protocol of Cardiff et al. [107]. The liver sections were cut and stained with hematoxylin and eosin, then examined microscopically for histopathological changes.

### 3.11. Statistical Analysis

The HPLC analyses, TPC, and in vitro antioxidant tests were performed in triplicate.

In vitro data were expressed as the mean ± standard deviation (SD), while the in vivo were expressed as mean ± standard error of the mean (SEM), using software XLSTAT version 2020.4. The Tukey post hoc test was used to analyze differences between groups after a one-way analysis of variance (ANOVA). A value of * *p* < 0.05 indicated significance, ** *p* ˂ 0.01 indicated high significance, and *** *p* ˂ 0.001 indicated very high statistical significance.

## 4. Conclusions

Our results show that our hydroethanolic *F. carica* fruit extracts, especially the *azendjar* variety, are rich in several phenolic compounds, with a dominance of rutin. In vitro, the extracts exerted a potent antioxidant capacity in both DPPH and FRAP assays, where the azendjar variety of the dark peel color indicated the strongest potency. In vivo tests have shown that the extracts helped to reduce the damages caused by carbon tetrachloride intoxication, presented by the increases in serum enzymes, the decreases in catalase activity, and histological damage to the liver tissues. These results confirm the antioxidant activity of the extracts and their potential to reduce the risk in some conditions connected with oxidative stress such as cancer, aging, and inflammatory diseases.

## Figures and Tables

**Table 1:** Values of determined chemical compounds of ethanolic extracts from Az and Ta varieties of *F. carica* fruits.

Component	Retention Time [min]	Mass Per 100 g of Fig DW [mg]				Mass Per 1 g of Fig Extract [µg]			
		Az		Ta		Az		Ta	
		Average	RSD [%]	Average	RSD [%]	Average	RSD [%]	Average	RSD [%]
3,4-dihydroxybenzoic acid	8.63	1.892	2.5	0.744	2.1	29.72	2.5	10.28	2.1
4-hydroxybenzoic acid	12.70	0.585	2.7	0.625	1.7	9.19	2.7	8.64	1.7
vanillic acid	14.95	2.771	3.5	1.761	3.9	43.51	3.5	24.34	3.9
rutin	28.88	8.957	2.0	2.544	0.8	140.64	2.0	35.16	0.8
rosmarinic acid	30.44	0.055	40.2			0.87	40.2		
cinnamic acid	33.93	0.049	4.3	0.100	9.3	0.77	4.3	1.38	9.3
quercetin	35.61	0.644	2.9	0.151	3.8	10.11	2.9	2.09	3.8

**Table 2:** Extraction yields, TPC, and antioxidant activity in vitro with DPPH and FRAP assays of F. carica fruit extracts.

Studied Parameter	Extraction Yields	Total Phenolic Content (TPC)		In Vitro Antioxidant Activity		
				DPPH Assay	FRAP Assay	
Unit of Measurement	%	mg GAE/100 g of Extract	mg GAE/100 g DW of Figs	IC 50 mg/mL	mmol Fe (II)/100 g of Extract	mmol Fe (II)/100 g DW of Figs
Az variety	10.15	403.66 ± 32.11 *	257.06 ± 20.44	0.417 ± 0.032 ^1^*** 0. 655 ± 0.050 ^2^***	31.55 ± 1.43 **	20.09 ± 0.91
Ta variety	9.95	339.44 ± 10.33	245.57 ± 7.47	0.582 ± 0.015 ^1^ 0.804 ± 0.021 ^2^	26.08 ± 0.66	18.86 ± 0.47
Ascorbic acid	/	/		0.0995 ^1^	/	

The data are expressed as a mean ± SD. ^1^ Result expressed per pure extract/substance, ^2^ result expressed per dry weight of figs, * *p* ˂ 0.05 indicates a statistically significant difference, ** *p* ˂ 0.01 indicates a highly statistically significant difference, and *** *p* ˂ 0.001 indicates a very highly statistically significant difference between the results of the Az variety and those of the Ta variety.

**Table 3:** Evolution of body weight of rats in the differently treated groups during the experiment. T-: (no intoxication, untreated); T+: (intoxicated, untreated); STD, Dose 1, and Dose 2 (intoxicated, treated with vitamin C 50 mg/kg, fig extracts 300 mg/kg, and 600 mg/kg bw, respectively).

Groups		Day 0	24 h after CCl_4_		Final Day	
		Weight [g]	Weight [g]	Growth Rate [%]	Weight [g]	Growth Rate [% ]
T-		160.8 ± 1.78	162.7 ± 1.48	1.18	164 ± 4.52	1.99
T+		207.4 ± 3.28	186.6 ± 3.36 ***	−10.02	190 ± 4.30 ***	−8.38
STD (Vitamin C)		169.8 ± 4.38	161.8 ± 2.77 **	−4.71	176.4 ± 7.50	3.88
Dose 1	Az	187.4 ± 1.14	181 ± 2.82 **	−3.41	197.2 ± 6.83 *	5.22
	Ta	167.8 ± 3.11	151.4 ± 5.03 ***	−9.77	163 ± 5.70	−2.86
Dose 2	Az	183.6 ± 3.43	177.00 ± 4.79 *	−3.59	192.4 ± 7.56 *	4.79
	Ta	171.4 ± 2.50	162.4 ± 5.02 **	−5.25	176 ± 6.81	2.68

Each value represents the mean ± SEM of five rats. * *p* < 0.05; ** *p* < 0.01; *** *p* < 0.001 significantly different compared to the first day.

**Table 4:** Determination of the hepatic biochemical parameters such as AST, ALT, BILT, BILD, and ALP.

Groups		Biochemical Parameter				
		AST [Ul/L]	ALT [Ul/L]	BILT [mg/L]	BILD [mg/L]	ALP [µL/L]
**T-**		82.0 ± 3.9 ***##	126.0 ± 9.1 ***###	1.23± 0.2 ***##	0.3 ± 0.1 ***#	180.2 ± 34.9 ***##
**T+ (CCl_4_)**		570.4 ± 26.5 ###	594.8 ± 38.5 ###	3.78 ± 0.3 ###	3.1 ± 0.3 ###	602.2 ± 43.6 ###
**STD (vit C)**		103.8 ± 13.4 ***	161.8 ± 5.4 ***	1.7 ± 0.1 ***	0.8 ± 0.4 ***	269.0 ± 17.6 *** δδ
**Dose 1** **(300mg/kg)**	**Az**	95.0 ± 7.0 *** δδ	132.6 ± 15.6 ***##	1.4 ± 0.2 ***#	0.7 ± 0.2 *** δδ	318.8 ± 4 ***# δδ
	**Ta**	118.4 ± 20.5 *** δδ ^a’^	150.6 ± 11.0 ***δδ	1.4 ± 0.2 ***#	0.8 ± 0.2 *** δδ	347.2 ± 16.1 ***### δδδ
**Dose 2** **(600mg/kg)**	**Az**	88.2 ± 9.3 ***	106.0 ± 9.1 ***δδ ###	0.8 ± 0.2 ***### δδ	0.6 ± 0.1 *** δδδ	239.4 ± 15.5 ***# δ
	**Ta**	91.4 ± 7.2 ***δ	115.8 ± 12.7 ***###	1.2 ± 0.1 ***### ^b’^	0.8 ± 0.2 *** δδ	241.6 ± 16.3 ***# δ

Each value represents the mean ± SEM of five rats. One symbol indicates *p* < 0.05; two symbols indicate *p* < 0.01; three symbols indicate *p* < 0.001 significantly different. Symbols: * compared to T+, # compared to STD, δ compared to T-, a’ is a comparison between groups treated with Dose 1 (300 mg/kg); b’ is a comparison between groups treated with Dose 2 (600 mg/kg).

## Data Availability

Data will be made available on request.

## Abbreviations

ANOVA	analysis of variance
ALT	alanine aminotransferase
ALP	alkaline phosphatase
AST	aspartate aminotransferase
ATT	acute toxicity test
Az	*azendjar*
BILD	bilirubin direct
BILT	bilirubin total
BSA	bovine serum albumin
bw	body weight
CAT	catalase
*GAE*	gallic acid equivalent
DPPH	2,2-diphenyl-1-picryl-hydrazyl
DW	dry weight
FCR	Folin–Ciocalteu reagent
FW	fresh weight
FRAP	ferric ion reducing antioxidant power
HPLC-DAD	high-performance liquid chromatography with diode array detection
IC_50_	half-maximal inhibitory concentration
IGG	intragastric gavage
IP	intraperitoneal
ITAFV	Technical Institute for Fruit Trees in Algiers, Algeria
MDA	malondialdehyde
NLFO	National Livestock Feed Office
OECD	Organization for Economic Cooperation and Development
ROS	reactive oxygen species
RSA	radical scavenging activity
SEM	standard error of the mean
SD	standard deviation
Ta	*taamriouth*
TBA	thiobarbituric acid
TCA	trichloroacetic acid
TE	Trolox equivalent
TPC	total phenolic content

## References

[1] Sak K. (2014). Dependence of DPPH Radical Scavenging Activity of Dietary Flavonoid Quercetin on Reaction Environment. Mini Rev. Med. Chem..

[2] Schieber M., Chandel N.S. (2014). ROS Function in Redox Signaling and Oxidative Stress. Curr. Biol..

[3] Huyut Z., Beydemir Ş., Gülçin İ. (2017). Antioxidant and Antiradical Properties of Selected Flavonoids and Phenolic Compounds. Biochem. Res. Int..

[4] Rahman M.M., Islam M.B., Biswas M., Khurshid Alam A.H.M. (2015). In Vitro Antioxidant and Free Radical Scavenging Activity of Different Parts of Tabebuia Pallida Growing in Bangladesh. BMC Res. Notes.

[5] Saha S., Verma R.J. (2016). Antioxidant Activity of Polyphenolic Extract of Terminalia Chebula Retzius Fruits. J. Taibah Univ. Sci..

[6] Aryal S., Baniya M.K., Danekhu K., Kunwar P., Gurung R., Koirala N. (2019). Total Phenolic Content, Flavonoid Content and Antioxidant Potential of Wild Vegetables from Western Nepal. Plants.

[7] Li J., O W., Li W., Jiang Z.-G., Ghanbari H. (2013). Oxidative Stress and Neurodegenerative Disorders. Int. J. Mol. Sci..

[8] Ebrahimzadeh M., Nabavi S., Nabavi S., Dehpour A. (2011). Antioxidant Activity of Hydroalcholic Extract of *Ferula gummosa Boiss* Roots. Eur. Rev. Med. Pharmacol. Sci..

[9] Arackal J.J., Parameshwari S. (2021). Identification of Antioxidant Activity and Shelf Life Assay of Avocado Fruit Pulp Incorporated Chapattis. Materials Today: Proceedings.

[10] Proteggente A.R., Pannala A.S., Paganga G., van Buren L., Wagner E., Wiseman S., Put F. (2002). van de Dacombe, C.; Rice-Evans, C.A. The Antioxidant Activity of Regularly Consumed Fruit and Vegetables Reflects Their Phenolic and Vitamin C Composition. Free Radic. Res..

[11] Wojdyło A., Nowicka P., Carbonell-Barrachina Á.A., Hernández F. (2016). Phenolic Compounds, Antioxidant and Antidiabetic Activity of Different Cultivars of *Ficus carica* L. Fruits. J. Funct. Foods.

[12] Aguiar J., Gonçalves J.L., Alves V.L., Câmara J.S. (2020). Chemical Fingerprint of Free Polyphenols and Antioxidant Activity in Dietary Fruits and Vegetables Using a Non-Targeted Approach Based on QuEChERS Ultrasound-Assisted Extraction Combined with UHPLC-PDA. Antioxidants.

[13] Lima G.P.P., Vianello F., Corrêa C.R., Campos R.A.d.S., Borguini M.G. (2014). Polyphenols in Fruits and Vegetables and Its Effect on Human Health. Food Nutr. Sci..

[14] Oliveira A.P., Valentão P., Pereira J.A., Silva B.M., Tavares F., Andrade P.B. (2009). *Ficus carica* L.: Metabolic and Biological Screening. Food Chem. Toxicol..

[15] Calani L., Bresciani L., Rodolfi M., Del Rio D., Petruccelli R., Faraloni C., Ganino T. (2022). Characterization of the (Poly)Phenolic Fraction of Fig Peel: Comparison among Twelve Cultivars Harvested in Tuscany. Plants.

[16] Mawa S., Husain K., Jantan I. (2013). *Ficus carica* L. (Moraceae): Phytochemistry, Traditional Uses and Biological Activities. Evid. Based Complement. Alternat. Med..

[17] Alzahrani M.Y., Alshaikhi A.I., Hazzazi J.S., Kurdi J.R., Ramadan M.F. (2024). Recent Insight on Nutritional Value, Active Phytochemicals, and Health-enhancing Characteristics of Fig (*Ficus carica*). Food Safe Health.

[18] Çalişkan O., Aytekin Polat A. (2011). Phytochemical and Antioxidant Properties of Selected Fig (*Ficus carica* L.) Accessions from the Eastern Mediterranean Region of Turkey. Sci. Hortic..

[19] Kebal L., Pokajewicz K., Djebli N., Mostefa N., Poliwoda A., Wieczorek P.P. (2022). HPLC-DAD Profile of Phenolic Compounds and In Vitro Antioxidant Activity of *Ficus carica* L. Fruits from Two Algerian Varieties. Biomed. Pharmacother..

[20] Pereira C., López-Corrales M., Serradilla M.J., Villalobos M.d.C., Ruiz-Moyano S., Martín A. (2017). Influence of Ripening Stage on Bioactive Compounds and Antioxidant Activity in Nine Fig (*Ficus carica* L.) Varieties Grown in Extremadura, Spain. J. Food Compost. Anal..

[21] Faleh E., Oliveira A.P., Valentão P., Ferchichi A., Silva B.M., Andrade P.B. (2012). Influence of Tunisian *Ficus carica* Fruit Variability in Phenolic Profiles and In Vitro Radical Scavenging Potential. Rev. Bras. Farmacogn..

[22] De Masi L., Vella F.M., Laratta B., Volpe M.G., Tiseo M., La Cara F. (2017). Biochemical and Genetic Characterization of a Red Fig Cultivar (*Ficus carica*) from Southern Italy. Acta Hortic..

[23] Soltana H., De Rosso M., Lazreg H., Vedova A.D., Hammami M., Flamini R. (2018). LC-QTOF Characterization of Non-Anthocyanic Flavonoids in Four Tunisian Fig Varieties. J. Mass. Spectrom..

[24] Trifunschi S., Munteanu M., Ardelean D., Orodan M., Osser G., Gligor R. (2015). Flavonoids and Polyphenols Content and Antioxidant Activity of *Ficus carica* L. Extracts from Romania. Zb. Mat. Srp. Prir. Nauk..

[25] Ganeshpurkar A., Saluja A.K. (2017). The Pharmacological Potential of Rutin. Saudi Pharm. J..

[26] Enogieru A.B., Haylett W., Hiss D.C., Bardien S., Ekpo O.E. (2018). Rutin as a Potent Antioxidant: Implications for Neurodegenerative Disorders. Oxid. Med. Cell Longev..

[27] Satari A., Ghasemi S., Habtemariam S., Asgharian S., Lorigooini Z. (2021). Rutin: A Flavonoid as an Effective Sensitizer for Anticancer Therapy; Insights into Multifaceted Mechanisms and Applicability for Combination Therapy. Evid. Based Complement. Alternat. Med..

[28] Salehi B., Machin L., Monzote L., Sharifi-Rad J., Ezzat S.M., Salem M.A., Merghany R.M., El Mahdy N.M., Kılıç C.S., Sytar O. (2020). Therapeutic Potential of Quercetin: New Insights and Perspectives for Human Health. ACS Omega.

[29] Viuda-Martos M., Barber X., Pérez-Álvarez J.A., Fernández-López J. (2015). Assessment of Chemical, Physico-Chemical, Techno-Functional and Antioxidant Properties of Fig (*Ficus carica* L.) Powder Co-Products. Ind. Crops Prod..

[30] Semaming Y., Pannengpetch P., Chattipakorn S.C., Chattipakorn N. (2015). Pharmacological Properties of Protocatechuic Acid and Its Potential Roles as Complementary Medicine. Evid. Based Complement. Alternat. Med..

[31] Veberic R., Colaric M., Stampar F. (2008). Phenolic Acids and Flavonoids of Fig Fruit (*Ficus carica* L.) in the Northern Mediterranean Region. Food Chem..

[32] Benmaghnia S., Meddah B., Tir-Touil A., Antonio Gabaldon Hernandez J. (2019). Phytochemical Analysis, Antioxidant and Antimicrobial Activities of Three Samples of Dried Figs (*Ficus carica* L.) from the Region of Mascara. J. Microb. Biotech. Food Sci..

[33] Soni N., Mehta S., Satpathy G., Gupta R.K. (2014). Estimation of Nutritional, Phytochemical, Antioxidant and Antibacterial Activity of Dried Fig (*Ficus carica*). J. Pharmacogn. Phytochem..

[34] Krakowska A., Rafińska K., Walczak J., Kowalkowski T., Buszewski B. (2017). Comparison of Various Extraction Techniques of Medicago Sativa: Yield, Antioxidant Activity, and Content of Phytochemical Constituents. J. AOAC Int..

[35] Lezoul N.E.H., Belkadi M., Habibi F., Guillén F. (2020). Extraction Processes with Several Solvents on Total Bioactive Compounds in Different Organs of Three Medicinal Plants. Molecules.

[36] Lasano N.F., Ramli N.S., Hamid A.H., Karim R., Pak Dek M.S., Shukri R. (2019). Effects of Different Extraction Solvents on Polyphenols and Antioxidant Capacity of Peel, Pulp and Seed Kernel of Kuini (*Mangifera odorata*). Orient. Pharm. Exp. Med..

[37] Katalinić V., Možina S.S., Skroza D., Generalić I., Abramovič H., Miloš M., Ljubenkov I., Piskernik S., Pezo I., Terpinc P. (2010). Polyphenolic Profile, Antioxidant Properties and Antimicrobial Activity of Grape Skin Extracts of 14 *Vitis vinifera* Varieties Grown in Dalmatia (Croatia). Food Chem..

[38] Mahmoudi S., Khali M., Mahmoudi N. (2013). Etude de l’extraction Des Composés Phénoliques de Différentes Parties de La Fleur d’artichaut (*Cynara scolymus* L.). Nat. Technol..

[39] Abdel-Rahman R., Ghoneimy E., Abdel-Wahab A., Eldeeb N., Salem M., Salama E., Ahmed T. (2021). The Therapeutic Effects of *Ficus carica* Extract as Antioxidant and Anticancer Agent. S. Afr. J. Bot..

[40] Bey M.B., Louaileche H. (2015). A Comparative Study of Phytochemical Profile and In Vitro Antioxidant Activities of Dark and Light Dried Fig (*Ficus carica* L.) Varieties. J. Phytopharmacol..

[41] Hoxha L., Kongoli R., Hoxha M. (2015). Antioxidant Activity of Some Dried Autochthonous Albanian Fig (*Ficus carica*) Cultivars. IJCST.

[42] Mopuri R., Ganjayi M., Meriga B., Koorbanally N.A., Islam M.S. (2018). The Effects of *Ficus carica* on the Activity of Enzymes Related to Metabolic Syndrome. J. Food Drug Anal..

[43] Nakilcioğlu-Taş E., Ötleş S. (2021). Influence of Extraction Solvents on the Polyphenol Contents, Compositions, and Antioxidant Capacities of Fig (*Ficus carica* L.) Seeds. An. Acad. Bras. Ciênc..

[44] Harzallah A., Bhouri A.M., Amri Z., Soltana H., Hammami M. (2016). Phytochemical Content and Antioxidant Activity of Different Fruit Parts Juices of Three Figs (*Ficus carica* L.) Varieties Grown in Tunisia. Ind. Crops Prod..

[45] Baliyan S., Mukherjee R., Priyadarshini A., Vibhuti A., Gupta A., Pandey R.P., Chang C.-M. (2022). Determination of Antioxidants by DPPH Radical Scavenging Activity and Quantitative Phytochemical Analysis of *Ficus religiosa*. Molecules.

[46] Palmeira L., Pereira C., Dias M.I., Abreu R.M.V., Corrêa R.C.G., Pires T.C.S.P., Alves M.J., Barros L., Ferreira I.C.F.R. (2019). Nutritional, Chemical and Bioactive Profiles of Different Parts of a Portuguese Common Fig (*Ficus carica* L.) Variety. Food Res. Int..

[47] Mujić I., Dudas S., Skutin H.M., Perusic D., Zeković Z., Lepojević Z., Radojković M., Vidović S., Milošević S., Mesic E.O. (2012). Determination of Antioxidant Properties of Fig Fruit Extracts (*Ficus carica* L.). Acta Hortic..

[48] Aljane F., Neily M.H., Msaddak A. (2020). Phytochemical Characteristics and Antioxidant Activity of Several Fig (*Ficus carica* L.) Ecotypes. Ital. J. Food Sci..

[49] Solomon A., Golubowicz S., Yablowicz Z., Grossman S., Bergman M., Gottlieb H.E., Altman A., Kerem Z., Flaishman M.A. (2006). Antioxidant Activities and Anthocyanin Content of Fresh Fruits of Common Fig ( *Ficus carica* L.). J. Agric. Food Chem..

[50] Martins N., Barros L., Ferreira I.C.F.R. (2016). In Vivo Antioxidant Activity of Phenolic Compounds: Facts and Gaps. Trends Food Sci. Technol..

[51] Hssaini L., Charafi J., Razouk R., Hernández F., Fauconnier M., Ennahli S., Hanine H. (2020). Assessment of Morphological Traits and Fruit Metabolites in Eleven Fig Varieties (*Ficus carica* L.). Int. J. Fruit. Sci..

[52] Pande G., Akoh C.C. (2010). Organic Acids, Antioxidant Capacity, Phenolic Content and Lipid Characterisation of Georgia-Grown Underutilized Fruit Crops. Food Chem..

[53] Truong H.N., Nguyen H.N., Nguyen T.K.N., Le M.H., Tran H.G., Huynh N., Van Nguyen T. (2014). Establishment of a Standardized Mouse Model of Hepatic Fibrosis for Biomedical Research. Biomed. Res. Ther..

[54] Dutta S., Chakraborty A.K., Dey P., Kar P., Guha P., Sen S., Kumar A., Sen A., Chaudhuri T.K. (2018). Amelioration of CCl_4_ Induced Liver Injury in Swiss Albino Mice by Antioxidant Rich Leaf Extract of *Croton bonplandianus Baill*. PLoS ONE.

[55] Zhang G., Wang X., Chung T.-Y., Ye W., Hodge L., Zhang L., Chng K., Xiao Y.-F., Wang Y.J. (2020). Carbon Tetrachloride (CCl_4_) Accelerated Development of Non-Alcoholic Fatty Liver Disease (NAFLD)/Steatohepatitis (NASH) in MS-NASH Mice Fed Western Diet Supplemented with Fructose (WDF). BMC Gastroenterol..

[56] Nemiche S., Ait Hamadouche N., Nemmiche S., Fauconnier M.-L., Tou A. (2022). Ameliorative or Corrective Effects of Fig “*Ficus carica*” Extract on Nickel-Induced Hepatotoxicity in Wistar Rats. Toxicol. Res..

[57] Ubhenin A.E., Igbe I., Adamude F.A., Falodun A. (2016). Hepatoprotective Effects of Ethanol Extract of Caesalpiniabonduc against Carbon Tetrachloride Induced Hepatotoxicity in Albino Rats. J. Appl. Sci. Environ. Manag..

[58] El-Hadary A.E., Elsanhoty R.M., Ramadan M.F. (2019). In Vivo Protective Effect of *Rosmarinus officinalis* Oil against Carbon Tetrachloride (CCl_4_)-Induced Hepatotoxicity in Rats. PharmaNutrition.

[59] Osadebe P.O., Okoye F.B., Uzor P.F., Nnamani N.R., Adiele I.E., Obiano N.C. (2012). Phytochemical Analysis, Hepatoprotective and Antioxidant Activity of *Alchornea cordifolia* Methanol Leaf Extract on Carbon Tetrachloride-Induced Hepatic Damage in Rats. Asian Pac. J. Trop. Med..

[60] Olayode O.A., Daniyan M.O., Olayiwola G. (2020). Biochemical, Hematological and Histopathological Evaluation of the Toxicity Potential of the Leaf Extract of *Stachytarpheta cayennensis* in Rats. J. Tradit. Complement. Med..

[61] Hira S., Gulfraz M., Saqlan Naqvi S.M., Qureshi R., Gul H., Shad I. (2021). Protective Effect of *Ficus carica* Fruit against Carbon Tetrachloride Induced Hepatic Toxicity in Mice. J. Anim. Plant Sci..

[62] Fouad D., Alhatem H., Abdel-Gaber R., Ataya F. (2019). Hepatotoxicity and Renal Toxicity Induced by Gamma-Radiation and the Modulatory Protective Effect of *Ficus carica* in Male Albino Rats. Res. Vet. Sci..

[63] Turan A., Celik I. (2016). Antioxidant and Hepatoprotective Properties of Dried Fig against Oxidative Stress and Hepatotoxicity in Rats. Int. J. Biol. Macromol..

[64] Unsal V., Cicek M., Sabancilar İ. (2021). Toxicity of Carbon Tetrachloride, Free Radicals and Role of Antioxidants. Rev. Environ. Health.

[65] Moreira P.R., Maioli M.A., Medeiros H.C., Guelfi M., Pereira F.T., Mingatto F.E. (2014). Protective Effect of Bixin on Carbon Tetrachloride-Induced Hepatotoxicity in Rats. Biol. Res..

[66] Molehin O.R., Oloyede O.I., Idowu K.A., Adeyanju A.A., Olowoyeye A.O., Tubi O.I., Komolafe O.E., Gold A.S. (2017). White Butterfly (*Clerodendrum volubile*) Leaf Extract Protects against Carbon Tetrachloride-Induced Hepatotoxicity in Rats. Biomed. Pharmacother..

[67] Adikwu E., Deo O. (2013). Hepatoprotective Effect of Vitamin C (Ascorbic Acid). Pharmacol. Pharm..

[68] Khalil I., Ghani M., Khan M.R., Akbar F. (2020). Evaluation of Biological Activities and In Vivo Amelioration of CCl_4_ Induced Toxicity in Lung and Kidney with Abutilon Pannosum (G. Forst.) Schltdl. in Rat. J. Ethnopharmacol..

[69] Naz I., Khan M.R., Zai J.A., Batool R., Zahra Z., Tahir A. (2020). Pilea Umbrosa Ameliorate CCl_4_ Induced Hepatic Injuries by Regulating Endoplasmic Reticulum Stress, pro-Inflammatory and Fibrosis Genes in Rat. Environ. Health Prev. Med..

[70] Ali S., Khan M.R., Iqbal J., Shah S.A., Abbasi B.A., Yaseen T., Batool R., Ali I., Hussain M.D., Kazi M. (2022). Chemical Characterization and Evaluation of the Nephroprotective Potential of Parrotiopsis Jacquemontiana (Decne) Rehder and Periploca Hydaspidis Falc Crude Extract in CCl_4_-Induced Male Sprague-Dawley Rats. Saudi J. Biol. Sci..

[71] Khadeer Ahamed M.B., Krishna V., Dandin C.J. (2010). In Vitro Antioxidant and In Vivo Prophylactic Effects of Two γ-Lactones Isolated from *Grewia tiliaefolia* against Hepatotoxicity in Carbon Tetrachloride Intoxicated Rats. Eur. J. Pharmacol..

[72] Bouabid K., Lamchouri F., Toufik H., Faouzi M.E.A. (2020). Phytochemical Investigation, In Vitro and In Vivo Antioxidant Properties of Aqueous and Organic Extracts of Toxic Plant: *Atractylis gummifera* L.. J. Ethnopharmacol..

[73] Pirinççioğlu M., Kızıl G., Kızıl M., Kanay Z., Ketani A. (2014). The Protective Role of Pomegranate Juice against Carbon Tetrachloride–Induced Oxidative Stress in Rats. Toxicol. Ind. Health.

[74] Elsawy H., Badr G.M., Sedky A., Abdallah B.M., Alzahrani A.M., Abdel-Moneim A.M. (2019). Rutin Ameliorates Carbon Tetrachloride (CCl_4_ )-Induced Hepatorenal Toxicity and Hypogonadism in Male Rats. PeerJ.

[75] Makni M., Chtourou Y., Fetoui H., Garoui E.M., Boudawara T., Zeghal N. (2011). Evaluation of the Antioxidant, Anti-Inflammatory and Hepatoprotective Properties of Vanillin in Carbon Tetrachloride-Treated Rats. Eur. J. Pharmacol..

[76] Foti M.C., Ingold K.U. (2003). Mechanism of Inhibition of Lipid Peroxidation by γ-Terpinene, an Unusual and Potentially Useful Hydrocarbon Antioxidant. J. Agric. Food Chem..

[77] Zhang L., Zhang J., Zang H., Yin Z., Guan P., Yu C., Shan A., Feng X. (2024). Dietary pterostilbene exerts potential protective effects by regulating lipid metabolism and enhancing antioxidant capacity on liver in broilers. J. Anim. Physiol. Anim. Nutr..

[78] Rudrapal M., Khairnar S.J., Khan J., Dukhyil A.B., Ansari M.A., Alomary M.N., Alshabrmi F.M., Palai S., Deb P.K., Devi R. (2022). Dietary Polyphenols and Their Role in Oxidative Stress-Induced Human Diseases: Insights Into Protective Effects, Antioxidant Potentials and Mechanism(s) of Action. Front. Pharmacol..

[79] Okoro I.O., Okoro E.O., Isoje F.E., Oyubu G. (2022). Protective Effects of Alstonia Congensis Methanolic Extract against CCl_4_ Induced Liver Damage in Wistar Rats. Sci. Afr..

[80] Timoumi R., Amara I., Neffati F., Najjar M.F., El Golli-Bennour E., Bacha H., Abid-Essefi S. (2019). Acute Triflumuron Exposure Induces Oxidative Stress Responses in Liver and Kidney of Balb/C Mice. Environ. Sci. Pollut. Res..

[81] Liu Y., Cao L., Du J., Jia R., Wang J., Xu P., Yin G. (2015). Protective Effects of Lycium Barbarum Polysaccharides against Carbon Tetrachloride-Induced Hepatotoxicity in Precision-Cut Liver Slices In Vitro and In Vivo in Common Carp (*Cyprinus carpio* L.). Comp. Biochem. Physiol. C Toxicol. Pharmacol..

[82] Duan Z., Zhang Y., Zhu C., Wu Y., Du B., Ji H. (2020). Structural Characterization of Phosphorylated Pleurotus Ostreatus Polysaccharide and Its Hepatoprotective Effect on Carbon Tetrachloride-Induced Liver Injury in Mice. Int. J. Biol. Macromol..

[83] Meng X., Wang Z., Liang S., Tang Z., Liu J., Xin Y., Kuang H., Wang Q. (2019). Hepatoprotective Effect of a Polysaccharide from Radix Cyathulae Officinalis Kuan against CCl_4_-Induced Acute Liver Injury in Rat. Int. J. Biol. Macromol..

[84] Aly A.A., Zaky E.A., Mahmoud H.A., Alrefaei A.F., Hameed A.M., Alessa H., Alsimaree A.A., Aljohani M., El-Bahy S.M., Kadasah S. (2021). The Impact of Addition Oats (*Avena sativa*) and Cinnamon on Cookies and Their Biological Effects on Rats Treated with Cirrhosis by CCL_4_. Saudi J. Biol. Sci..

[85] Al Doghaither H.A., Al-Sohaibani R.M., Omar U.M., Alharbi H.A. (2021). Biochemical and Histological Effects of Five Weeks Ingestion of Zamzam Water on the Liver and Kidneys of Wistar Rats. Saudi Pharm. J..

[86] Ojeaburu S.I., Oriakhi K. (2021). Hepatoprotective, Antioxidant and, Anti-Inflammatory Potentials of Gallic Acid in Carbon Tetrachloride-Induced Hepatic Damage in Wistar Rats. Toxicol. Rep..

[87] Ozturk I.C., Ozturk F., Gul M., Ates B., Cetin A. (2009). Protective Effects of Ascorbic Acid on Hepatotoxicity and Oxidative Stress Caused by Carbon Tetrachloride in the Liver of Wistar Rats. Cell Biochem. Funct..

[88] Slatnar A., Klancar U., Stampar F., Veberic R. (2011). Effect of Drying of Figs (*Ficus carica* L.) on the Contents of Sugars, Organic Acids, and Phenolic Compounds. J. Agric. Food Chem..

[89] Gilani A.H., Mehmood M.H., Janbaz K.H., Khan A., Saeed S.A. (2008). Ethnopharmacological Studies on Antispasmodic and Antiplatelet Activities of *Ficus carica*. J. Ethnopharmacol..

[90] Mustafa K., Yu S., Zhang W., Mohamed H., Naz T., Xiao H., Liu Y., Nazir Y., Fazili A.B.A., Nosheen S. (2021). Screening, Characterization, and in Vitro-ROS Dependent Cytotoxic Potential of Extract from *Ficus carica* against Hepatocellular (HepG2) Carcinoma Cells. S. Afr. J. Bot..

[91] Singleton V.L., Rossi J.A. (1965). Colorimetry of Total Phenolics with Phosphomolybdic-Phosphotungstic Acid Reagents. Am. J. Enol. Vitic..

[92] Atoui A. (2005). Tea and Herbal Infusions: Their Antioxidant Activity and Phenolic Profile. Food Chem..

[93] Benzie I.F.F., Strain J.J. (1996). The Ferric Reducing Ability of Plasma (FRAP) as a Measure of “Antioxidant Power”: The FRAP Assay. Anal. Biochem..

[94] Pulido R., Bravo L., Saura-Calixto F. (2000). Antioxidant Activity of Dietary Polyphenols As Determined by a Modified Ferric Reducing/Antioxidant Power Assay. J. Agric. Food Chem..

[95] OECD (2022). Test No. 425: Acute Oral Toxicity: Up-and-Down Procedure.

[96] Loomis T.A., Hayes W.A. (1996). Loomis’s Essentials of Toxicology.

[97] Ashraf G.M., Alghamdi B.S., Alshehri F.S., Alam M.Z., Tayeb H.O., Tarazi F.I. (2021). Empagliflozin Effectively Attenuates Olanzapine-Induced Body Weight Gain in Female Wistar Rats. Front. Pharmacol..

[98] Murray R.L. (1984). Aspartate Aminotransferase. Clinical Chemistry: Theory, Analysis and Correlation.

[99] Murray R.L. (1984). Alanine Aminotransferase. Clinical Chemistry: Theory, Analysis and Correlation.

[100] Murao S., Tanaka N. (1981). A New Enzyme “Bilirubin Oxidase” Produced by *Myrothecium verrucaria* MT-1. Agric. Biol. Chem..

[101] Garber C.C. (1981). Jendrassik--Grof Analysis for Total and Direct Bilirubin in Serum with a Centrifugal Analyzer. Clin. Chem..

[102] Wenger C., Kaplan L.A., Pesce A.J. (1984). Alkaline Phosphatase. Clinical Chemistry. Theory, Analysis and Correlation.

[103] Boni A.N.R., Kouassi K., Ayebe A.E., Yapi H.F., Djaman A.J., Nguessan J.D. (2015). In Vivo Antioxidant Activity of Methanolic Extract of Stem Bark of *Spondias mombin* L. on Carbon Tetrachloride Induced Oxidative Stressin Wistar Rats. J. Chem. Pharm. Res..

[104] Bradford M.M. (1976). A Rapid and Sensitive Method for the Quantitation of Microgram Quantities of Protein Utilizing the Principle of Protein-Dye Binding. Anal. Biochem..

[105] Draper H.H., Hadley M. (1990). Malondialdehyde Determination as Index of Lipid Peroxidation. Methods in Enzymology.

[106] Claiborne A. (1985). Catalase Activity. Handbook of Methods for Oxygen Radical Research.

[107] Cardiff R.D., Miller C.H., Munn R.J. (2014). Manual Hematoxylin and Eosin Staining of Mouse Tissue Sections. Cold Spring Harb. Protoc..

